# Music Therapy for Reduction of Anhedonia: Protocol for a Sequential Mixed Methods Pre-Post Pilot Study

**DOI:** 10.2196/90667

**Published:** 2026-09-23

**Authors:** Katrina Skewes McFerran, Alec John Jamieson, Yasmin Blunck, Rebecca Glarin, Jamie Elliott, Kirsten Byrony Hillman, Lucy Elizabeth Bolger

**Affiliations:** 1 Faculty of Fine Arts and Music, The University of Melbourne, St Kilda Road, Southbank, Victoria, 3006, Australia, 61 407350251; 2 Department of Psychiatry, The University of Melbourne, Parkville, Victoria, Australia; 3 Department of Biomedical Engineering, The University of Melbourne, Parkville, Victoria, Australia; 4 Melbourne Brain Centre Imaging Unit, The University of Melbourne, Parkville, Victoria, Australia; 5 Department of Radiology, The University of Melbourne, Parkville, Victoria, Australia

**Keywords:** music therapy, anhedonia, pleasure, musical pleasure, functional magnetic resonance imaging, fMRI, reward liking

## Abstract

**Background:**

The transdiagnostic symptom of anhedonia, or the loss of pleasure in previously pleasurable things, is common across several chronic physical and mental illnesses. Music therapy has been effective in treating a range of conditions associated with anhedonia, but only 3 cases have been reported that targeted increasing hedonic capacity and all relied on descriptive data (qualitative and quantitative). Functional magnetic resonance imaging (fMRI) evidence demonstrates that pleasure is associated with increased activity in the limbic system, including the anterior cingulate cortex, as well as the prefrontal cortex. As such, changes within these regions may provide objective evidence to explore how music therapy acts to reduce anhedonia symptomatology.

**Objective:**

Our primary aim is to examine the feasibility of a music therapy intervention to increase hedonic capacity among individuals with anhedonia. We also aim to examine congruence across psychometric results and interview data to generate estimates of effect size and explore participants’ qualitative experiences. In addition, we aim to identify any correspondence between these outcomes and changes in brain activity associated with reward processing during targeted fMRI tasks. This study will generate hypotheses for testing in future studies.

**Methods:**

This pilot study uses a sequential mixed methods design to first identify whether there are pre-post changes in the primary outcome measures and then to identify correlations between interview data, psychometric data, and objective measures of brain structure and functioning (fMRI). Participants were 13 adults with high cognitive function who scored within the clinical range for anhedonia on the Dimensional Anhedonia Rating Scale (DARS). Participants were engaged in a 4-month music therapy intervention developed through preliminary case studies, which involved daily independent pleasure-focused music activities and regular contact with a therapeutic guide. The fMRI data acquired include resting-state fMRI and task-based functional paradigms designed to probe musical pleasure (music listening task) and reward prediction (reversal reward learning task). Data will be analyzed using individual general linear models to identify associations with symptom improvement, as well as longitudinal changes in functioning.

**Results:**

The study received internal funding support from the University of Melbourne in September 2024. Data collection commenced in August 2025 and was concluded by mid-2026. As of September 2026, 13 participants have been recruited, and data will be analyzed by the end of 2026, with results expected to be published in 2027.

**Conclusions:**

The results of this pilot study will contribute to knowledge about the effects of this music therapy intervention on reducing anhedonia and introduce a study protocol that could be used in subsequent, more robust studies to explore potential mechanisms.

## Introduction

### Background

Anhedonia has been recognized as a transdiagnostic symptom associated with a range of chronic illnesses [1]. It is defined as “markedly diminished interest or pleasure in all, or almost all, activities” [2] and is often accompanied by a lack of motivation or engagement and diminished decision-making capacity. Although it is a core symptom of major depressive disorder (MDD), it is also associated with schizophrenia, substance use disorder, posttraumatic stress disorder, as well as neurological conditions such as Parkinson disease, traumatic brain injury, and chronic pain [3-5]. Importantly, anhedonia symptoms are both clinically underrecognized [6] and respond poorly to conventional antidepressant treatments [7,8], highlighting an unmet need for effective interventions. Regarding psychological interventions, positive affect treatments that increase reward sensitivity are superior to those focusing on negative affect [9] and show some potential for novel treatments such as those using music.

A range of studies have shown music therapy to be effective in increasing positive affect in the above illnesses. For example, a Cochrane review of music therapy for people with schizophrenia [10] found a positive effect on global state and negative symptoms (which align with positive affect treatments). Another Cochrane review of music therapy for depression [11] found improvements in depression, anxiety, and daily functioning, and improvements in anxiety and depression were also demonstrated in a Cochrane review of patients with cancer [12]. A more recent systematic review of music therapy in Parkinson disease [13] suggested emotional and motivational improvements that may be related to anhedonia but were not specified as such. Similarly, a Cochrane review of music therapy for substance use disorders [14] identified a moderate effect on motivation for treatment or change when compared to standard care. Additional systematic reviews of music therapy demonstrate reasonably consistent effects on transdiagnostic symptoms such as anxiety [15], depression, and quality of life [16]. However, there is little understanding of the mechanisms underpinning music therapy interventions [17]. Anhedonia may be another transdiagnostic symptom worthy of investigation.

Music therapy has been described as a complex intervention that requires contextual sensitivity [18]. Complex interventions, however, do not typically operate via a single causal mechanism; instead, they involve a mixture of biophysiological, neuropsychological, and sociobehavioral mechanisms of action, influenced by an interplay of therapeutic factors [19]. Flow has been proposed as 1 possible neuropsychological mechanism of change that provides a positive framing of music therapy [20] and may be consistent with the idea of positive affect treatments. Our previous case studies in anhedonia introduce another possibility: an increased focus on pleasure [21,22]. As such, an increase in pleasurable experiences through music therapy may improve positive affect (reward liking) as well as motivation (reward wanting). There is surprisingly little literature in music therapy on the topic of pleasure [23]. However, reviews are emerging based on qualitative reports [24] and brain imaging studies [25].

There is a rich body of literature documenting the use of functional neuroimaging to investigate neural correlates of music listening. This work began with electroencephalographic measures [26], which demonstrated the ability of music to modulate cortical rhythms but was limited by the low spatial resolution of the technique. Throughout the 1990s, positron emission tomography (PET) imaging became the primary modality and was beneficial because of its ability to localize neural responses to cerebral regions. One landmark PET imaging study [27] found spontaneous activation (inferred from cerebral perfusion, which was visualized by an oxygen-15 radiotracer) of the limbic and paralimbic systems while passively listening to unfamiliar but reportedly “pleasurable” instrumental music. Specific areas of activation included the subcallosal cingulate gyrus, anterior cingulate cortex, hippocampus, anterior insula, and nucleus accumbens. These findings have been frequently replicated and expanded upon since the development of blood oxygenation level–dependent (BOLD) functional magnetic resonance imaging (fMRI) [28-31], which has identified further activations (particularly in response to specifically “pleasurable” music) in the inferior frontal gyrus, ventral striatum, and Rolandic operculum, for example. Comprehensive investigations of pleasure have identified an evolutionarily conserved hedonic network encompassing the ventral pallidum, nucleus accumbens, insula, and ventromedial prefrontal cortex [32,33]. Meta-analytic fMRI studies further demonstrate modality-specific representations, with the insula playing a key role in representing pleasure induced by music [33].

These findings about pleasure-induced responses are further supported by results in clinical populations. For example, 1 study [34] investigated the neural response to passive listening to personalized “neutral” and “enjoyable” music in 16 patients with MDD, compared to 15 controls. Both groups demonstrated greater BOLD activation in the medial orbitofrontal cortex and nucleus accumbens while listening to “enjoyable” compared to “neutral” music, with controls showing a higher magnitude of activation difference than patients in the medial orbitofrontal cortex. Similarly, reduced activation in the anterior cingulate cortex in response to pleasurable music and positive emotional face presentation was identified in a study investigating patients with remitted depression compared to matched controls [35].

These findings are informative but not fully representative of an anhedonia-specific investigative lens. Despite the vast array of fMRI studies focused on depression, there is limited research into anhedonia specifically. The existing literature has mainly used in-scanner reward tasks to probe functional activation and has identified correlations between anhedonia severity and ventral striatum or amygdala hypoactivity [36,37] and ventromedial prefrontal cortex hyperactivity [37]. A greater breadth of studies has investigated task-induced reward “wanting” and “liking” in MDD without specific correlation with reported anhedonia severity. These have reported increased recruitment of the paralimbic anterior cingulate cortex, dorsolateral prefrontal cortex, and medial prefrontal cortex during reward “wanting” and “liking” phases by patients [38,39], alongside hypoactivity of the limbic ventral striatum and nucleus accumbens, amygdala, and caudate, among others [7].

More recently, studies have focused on the specific concept of “musical anhedonia,” or the absence or reduction of pleasurable emotional arousal while listening to music [40,41]. People experiencing musical anhedonia have shown significant hypoactivity in the nucleus accumbens while listening to music, as well as decreased functional connectivity between the right superior temporal gyrus and the nucleus accumbens [42], evidencing disruptions in the connectivity of auditory sensory regions and the associated limbic structures that relate to the ability to generate the feeling of pleasure. This is a promising area of research but is distinct from the focus of this study, which is on general anhedonia with music as an intervention, and none of the participants reported changes specific to musical pleasure.

Despite the frequent occurrence of anhedonia associated with a range of chronic physical and mental illnesses, there is limited evidence of successful interventions. To date, only positive affect treatments have demonstrated repeated success [43], and the Craske model [9] informed the conceptualization of the music therapy intervention tested here, which includes the core features of regularity, daily practice, and cognitive attention to pleasurable sensations. To date, this music therapy intervention has resulted in measurable improvements in anhedonia symptoms (Dimensional Anhedonia Rating Scale [DARS]) for 3 case studies, as consolidated by interview data [21,22]. However, it is unclear whether these results will be repeated with a larger sample and whether further objective markers of improvement could be demonstrated. Therefore, pilot study data are needed to refine a focus on relevant dimensions of anhedonia that might be modified by a complex intervention such as music therapy through the exploration of objective measures of both anhedonia and pleasure. This manuscript serves as a published protocol for a pilot study and can be used in a subsequent study if findings indicate feasibility.

### Objectives

Our primary aim is to examine the feasibility of leveraging a music therapy intervention to increase hedonic capacity among individuals with anhedonia. We also aim to examine congruence across psychometric results and interview data to generate estimates of effect size and qualitative experience. In addition, we aim to identify any correspondence between these outcomes and changes in brain activity associated with reward processing during targeted fMRI tasks. This study will generate hypotheses for testing in future studies by investigating the following research questions (RQs):

- RQ 1: To what extent is the music therapy intervention feasible, based on measures of participant adherence, engagement, and retention?
- RQ 2: Do overall changes in participant scores on psychometric measures of anhedonia from before to after the music therapy intervention correspond with patterns in descriptions of change in interviews?
- RQ 3: Are pre-post changes in activity across reward processing regions during fMRI tasks associated with changes in anhedonia symptoms?

### Study Design

Given the findings from previous case studies, a sequential mixed methods study design is suitable for use in this pre-post pilot study to explore congruences between psychometric, interview, and fMRI data regarding changes in hedonic capacity among individuals with anhedonia following the music therapy intervention. Neither a strictly explanatory nor exploratory model (according to Creswell and Plano [44] definitions) is used, but the design will prioritize quantitative data from psychometric scales and seek to understand any significant differences from before to after intervention using interview data collected after intervention. This will cautiously extend case study findings of the same music therapy intervention [21,22] to determine whether psychometric outcomes are significant at the group level, as well as to explore patterns in the interview data to better understand any changes on the 2 different outcome measures—the DARS [45] and the Snaith-Hamilton Pleasure Scale (SHAPS) [46]. It would be premature to include a control condition at this stage, and similarly, the sampling of participants will be restricted to high-functioning people with anhedonia to better understand the phenomenon before extending the research to more clinical populations, such as those with schizophrenia or MDD. In addition, the study will assess the feasibility of the intervention and study procedure to inform a subsequent larger-scale study [47]. Feasibility measures will evaluate the acceptability, adherence, and practicality of delivering the intervention in adults with high cognitive function, while preliminary estimates of intervention effects and variability will be used to inform sample size calculations and the design of a future fully powered study.

Several psychometric measures of anhedonia could be used to identify eligible participants for this study, with the DARS being most relevant because it includes a multidimensional focus on both anticipatory and consummatory pleasure that were measured in the previous case studies and appeared appropriate [21,22]. It has also demonstrated greater sensitivity to subtle differences in a German study of a similar cohort of young adults [48] and is more personalized, with participants listing chosen activities rather than having fixed items presented. By comparison, the SHAPS is more focused on consummatory pleasure [46] and has been widely validated in clinical populations (populations with schizophrenia, substance use, or depression) [49,50], which may be relevant for subsequent studies, making it useful for inclusion as a measure of change. Despite these differences, the DARS has strong convergent validity with the SHAPS in some research [51], and this may prove useful for exploring convergence with patterns from interview data, as well as correspondence with reward processing activity.

Subsequently, task-based fMRI data will be useful to explore whether activity in reward-related regions of the brain corresponds with changes in our outcomes of interest. These data will provide an estimate of effect size and identify key regions of interest to be explored in future studies.

## Methods

### Study Setting

Participants were recruited from within the authors’ research-led university, with advertisements being shared through formal and informal communication channels. The intention was to recruit participants who were able to complete the psychometric measures, commit to a daily musical activity routine, meet regularly with the music therapist to discuss practice experiences and plan for subsequent practices, and reflect on and provide subjective feedback on the intervention after it was concluded. Given these high demands, the study targeted staff and PhD students, and expectations were negotiated transparently before commencement.

### Ethical Considerations

This study was approved by the University of Melbourne Humanities, Arts, and Social Sciences (HASS) 2 Human Ethics Committee (27796), and plain language information was provided explaining the full extent of required participation and the limits of the evidence. Participation was not incentivized with payments, but the potential to participate in a novel intervention potentially motivated participation since there is minimal treatment available. Extended written consent was received from all participants, meaning that data or information used in this research project may also be used in future projects that are closely related to this project, are in the same general area, or could make valuable use of this data. Participant privacy and confidentiality were protected by processes of deidentification of data using a simple coding system, with all identifying information being stored in a password-protected file accessible only to the researchers.

### Eligibility Criteria

To identify participants with anhedonia, communications included a link to an online screening survey that prospective participants could independently access, comprising the DARS and questions assessing the inclusion criteria. The research team reviewed all survey responses, and eligible prospective participants were identified as scoring <44 on the DARS [45], based on the group differences identified between patients with major depressive episodes and healthy controls. This score has also been used in a study of university students where none of the healthy controls scored <44 points on the total scale [52]. All participants were required to complete a magnetic resonance imaging (MRI) safety implant checklist and be screened by qualified radiographers to deem them compatible with an MRI scan.

The inclusion criteria are being a professional, research, or academic member of the university community, having the cognitive capacity to select and discuss pleasure-inducing music, being aged >18 years, scoring <44 on DARS, agreeing to participate and having the capacity to provide informed consent, having a self-identified interest in music, and having English literacy skills suitable for participation in interviews. Exclusion criteria for all groups are failure to meet scanning requirements, including having metallic objects in the body (eg, a cardiac pacemaker or cochlear implant), a history of claustrophobia, permanent metal dental appliances, or a body weight ≥120 kg, or having a diagnosis of schizophrenia or MDD.

### Intervention

The music therapy intervention involves a 4-month process of therapeutic guiding through engagement with daily pleasure-focused music activities, developed through preliminary case studies in which a qualified music therapist works collaboratively with a participant to support and scaffold their process [21,22].

The therapist meets each participant weekly, which gradually tapers off after 8 weeks toward the closure of each participant’s process depending on their needs. These weekly meetings are typically 30 to 60 minutes long and designed to offer a supportive space where participants can share and reflect on experiences in their lives related to anhedonia and explore the ways they relate to pleasure in music. The meetings are also used to discuss the pragmatic aspects of daily pleasure-focused music engagement and collaboratively develop plans, with the intention of removing anhedonia-related barriers to participation. The process is tailored and flexible to the participant’s presenting needs and interests and may vary considerably depending on a range of factors, including but not limited to the level of support needed to identify pleasure-inducing music and music activities, needs related to safe engagement with emotionally potent music, and the degree to which the setting and context surrounding the activities create barriers or facilitate engagement.

Daily music activities of approximately 5 minutes are planned with each participant. This often begins with music listening due to ease of access and can expand to more active engagement, such as playing instruments, singing, or moving to music. Activities are flexibly tailored each week according to the participants’ emergent process and are designed to reduce identified barriers to promote their access to pleasurable feelings during these activities. Participants are also invited to complete a brief daily survey before and after their music activity. This includes pre-post ratings (from 1 to 10) of their anticipation and experience of pleasure during the activity, and a space for brief descriptive reflection. This aspect of the intervention is not compulsory but aims to invite participants’ cognitive engagement with their daily pleasure experience.

In addition to weekly meetings, participants also receive daily prompts via SMS or WhatsApp text message for the first 8 weeks from the music therapist to remind them of their plan for that day. These texts may draw on the words the person uses to describe their feelings of joy or pleasure in music and may include links to music where this reduces access barriers. An example of a SMS or WhatsApp text message reads as follows:

Hi [participant’s name], I hope your day is starting out well. Just a reminder for your pleasure-focussed music activity: listening to a song during your mid-morning break and tuning in to any feelings of joy, playfulness or creativity that might arise. [Song link provided]. Happy listening!

Closure of the intervention process is collaboratively planned, typically in the second half of the 4-month process, where participants are supported to increase their independence and autonomy in daily pleasure-focused music activities. This occurs through a gradual reduction of therapeutic scaffolding. Resources may be developed with the participant in the closure phase to support continued engagement with pleasure-focused music activities, for example, developing playlists of pleasure-inducing tracks used successfully during the process.

### Outcomes

Feasibility will be primarily assessed in terms of participant adherence, engagement, and retention. Adherence will be evaluated by tracking participant attendance and the number and proportion of intervention sessions completed, with reasons for nonadherence recorded to determine whether these are related to the intervention or other factors. Engagement will be assessed by documenting completion of each component of the intervention, including participation in music therapy activities and any prescribed between-session practice, alongside interventionist ratings of participant engagement and proficiency where applicable. Participants will also be asked to provide brief open-ended feedback regarding their ability to engage with the intervention and any difficulties encountered. Retention will be assessed by recording the number and proportion of participants who withdraw from the study and those who complete the final assessment, with reasons for dropout documented. These measures will be used to determine whether the intervention can be delivered as intended, identify barriers to participation, and establish whether adherence and retention rates support progression to a larger definitive trial.

At baseline, participants complete an intake interview wherein demographic information including personal background and role within the university is recorded, as well as relevant information relating to participants’ general physical and mental health, experience of pleasure in daily life, any specific loss of pleasure in music, pleasure in preferred activities and lifestyle, and their historical and current relationship to music and music activity. A descriptive baseline of participants’ current level of hedonic capacity, including current experience of pleasure, enjoyment, social engagement, motivation, and decision-making capacity, is also recorded. These topics are revisited in a postintervention exit interview to capture perceived changes in pleasure experience and lifestyle associated with increased hedonic capacity; participant experience and characterization of musical pleasure through the intervention process; and participant experience of the intervention. Interview transcripts are generated for qualitative analysis. For interview questions, refer to Multimedia Appendix 1.

Pre-post DARS scores are recorded during the screening survey and prior to the exit interview. This timing allows the scale to be used to determine participant eligibility for the study. Pre-post SHAPS scores are recorded on fMRI scanning days, as part of the prescan briefing process. This timing provides an indicator of hedonic capacity at the time of scanning. Pre-post score comparisons from these validated tools will also be used to assess changes in anhedonia severity in individual participants after the intervention.

Scanning sessions for fMRI data collection will be conducted on a 7T MAGNETOM Plus MRI system (Siemens Healthineers) located at the Melbourne Brain Centre Imaging Unit (MBCIU) at the University of Melbourne. Imaging will consist of (1) a structural scan, (2) resting-state fMRI, and (3) BOLD fMRI, using 4 tasks: reward reversal, music, naturalistic positive music, and naturalistic neutral music listening. These tasks have been specifically developed to target reward- and music-processing–related neurocircuitry, which are hypothesized to be relevant to our intervention.

### Participant Timeline

**Figure 1.**
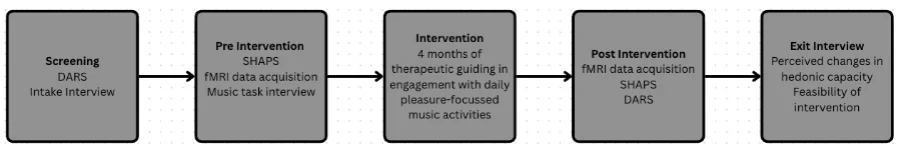
Schedule for data collection. DARS: Dimensional Anhedonia Rating Scale; fMRI: functional magnetic resonance imaging; SHAPS: Snaith-Hamilton Pleasure Scale.

The schedule for data collection is outlined in Figure 1. The DARS is independently completed online by participants who volunteer to participate. Questions on the online platform also confirm that they are professional, research, or academic members of the university community, aged >18 years, do not have a diagnosis of schizophrenia or MDD, and are interested in music. If these questions are confirmed and their score is <44 on the DARS, they are contacted by the research team to complete an intake interview, where this information is confirmed along with their English literacy skills, reflective cognitive capacity, and understanding of the requirements of the study. Times are then scheduled at the study center for further preintervention testing, including completion of the SHAPS and fMRI data acquisition. Brief interviews are conducted following the data acquisition to identify any feasibility issues with the music task. Following scans, participants commence 4 months of weekly meetings with the interventionist and undertake the daily music activities. Following completion of the intervention, participants return to the study center to complete the posttest measures of the DARS, SHAPS, and fMRI data acquisition and then participate in an exit interview.

### Sample Size

A sample size of 13 was selected to provide adequate depth of qualitative data (interviews) while maintaining feasibility for resource-intensive fMRI data collection. This sample size is sufficient for exploratory mixed methods analysis and effect size estimation, although it is underpowered for statistical inference [53].

### Imaging Protocol

MRI acquisition will be performed on a 7T Plus research scanner equipped with a 1Tx/32Rx channel head coil (Nova Medical Inc). A high-resolution structural T1-weighted image will be obtained from each participant using a magnetization-prepared 2 rapid acquisition gradient echoes (MP2RAGE [54]) sequence for coregistration with the functional images (parallel reduction factor 4, repetition time 5 s, echo time 2.04 ms, and flip angle 4° and 5°) in a 24 cm field of view, with a 330×330 pixel matrix, in-plane voxel size of 0.75×0.75 mm, a slice thickness of 0.75 mm, and 224 sagittal slices aligned parallel to the midline. To minimize head movement during scanning, foam pads will be inserted on either side of participants’ heads. Respiration and cardiac pulse will be recorded during the session at 50 Hz and 200 Hz, respectively, using a respiratory belt (Siemens) and pulse oximeter (Siemens) to be used for physiological noise correction. We will provide participants with insert earphones (Sensimetrics) to reduce acoustic noise from the MRI scanner as well as to deliver an audible-quality acoustic stimulus.

Functional (T2*-weighted) images will be obtained using a multiband and GRAPPA (generalized autocalibrating partially parallel acquisition)-accelerated gradient echo planar imaging sequence [55] in the steady state (multiband factor 6, parallel acceleration factor 2, repetition time 800 ms, echo time 22.2 ms, and flip angle 45°) in a 20.8 cm field of view with an isotropic 1.6 mm in-plane resolution and a slice thickness of 1.6 mm (no gap), with the phase-encoding direction from anterior to posterior. Eighty-four interleaved slices will be acquired parallel to the anterior-posterior commissure line, covering the whole brain. We will collect data for a resting-state scan and 4 functional sequences, as well as a reverse-phase dataset to be used for distortion correction.

The resting-state scan will be acquired over 5 minutes and 20 seconds. We will instruct participants to keep their eyes closed but not to fall asleep for the duration of the scan.

### Functional Imaging Paradigms

#### Reward-Reversal Task

This task is a previously described reversal learning paradigm [56] that aims to characterize reward prediction errors. In brief, we will present participants with a pair of target neutral stimuli (shapes) at the top and bottom of the screen. In each trial, 2 conditions can occur: either the top stimulus will be highlighted, or the bottom stimulus will be highlighted. Participants must predict whether the highlighted stimulus is associated with a reward or a punishment. If participants guess correctly, we will provide feedback with a green thumbs-up in the center of the screen, a pleasant, high-pitched tone, and a notification that they were correct. If they guess incorrectly, we will provide feedback with a red thumbs-down in the center of the screen, a low-pitched tone, and a notification that they were incorrect. Once a participant has reached a pseudorandom learning criterion of between 3 and 6 correct trials in a row, the reward-punishment association of the stimuli will be reversed. After each reversal, the learning criterion will be reset to a pseudorandom number between 3 and 6. The reversal will occur after each subsequent learning criterion is reached for the duration of the task. The total duration of this task is 13 minutes.

#### Music Task

Prior to scanning, participants self-select 5 pleasure-inducing tracks and nominate their most pleasurable 30-second excerpt of each track. Participants then identify 5 tracks with similar musical characteristics that they feel neutral listening to, using the following generative AI prompt:

What are five music recommendations with the same genre, instrumentation, singer gender, range, rhythmic energy, dynamic range as <insert pleasure-inducing track name here>?

This prescan process generates 10×30 seconds music excerpts (5×pleasure-inducing excerpts and 5×neutral excerpts). The music itself was not generated by AI but only identified using the AI prompt.

Within the music task, participants listen to each of the generated music excerpts twice, for a total of 20 × 30 seconds music excerpts (a similar time duration to foundational music chills research [57]), with a 15-second white noise break between each excerpt, running for 15 minutes in total. Participants are directed to close their eyes throughout the task and pay attention to any feelings that arise as they listen. The presentation of excerpts alternates between pleasure-inducing and neutral excerpts (or the reverse), and the order of excerpts is randomized within this alternating framework. All 5 excerpts of each type (pleasure-inducing or neutral) are played once before the sequence repeats a second time in the same order. Participants are manually allocated to start with the neutral or pleasure-inducing excerpts for this task, with the allocation evenly distributed across study participants.

#### Positive and Neutral Naturalistic Music Tasks

Participants will undergo 2 fMRI tasks with continuous music listening. They will preselect 1 of the 5 pleasure-inducing tracks and will listen to it in its entirety on loop for 5 minutes. They will then perform the same fMRI scan but with a corresponding neutral song played in its entirety on loop for 5 minutes.

### Data Analysis

Repeated-measures *t* tests will be conducted on pre-post DARS and SHAPS scores to examine directional change. Hedges *g* will be calculated to estimate effect sizes, which will inform power calculations for a future fully powered study. *P* values will be reported descriptively but not interpreted as indicators of statistical significance, given the small sample size (N=13).

fMRI data will be analyzed using individual general linear models to identify longitudinal changes associated with the intervention. For each task, blocked-design general linear model analyses will be specified at an individual participant level in Statistical Parametric Mapping version 12 (SPM12) to model specific conditions of interest. For the reward-reversal task, this will involve contrasting unexpected punishment trials with expected reward trials. For the music task, the primary contrast will compare pleasurable music with neutral music. Repeated-measures, flexible factorial designs (within-subject factors: primary contrast×time) will be used at the group level to examine longitudinal changes. These will then inform exploratory investigations of associations between these effects and symptom improvement.

Inductive thematic analysis will be used to identify patterns in descriptions of change and group similar ideas from across the interview data [58]. Labels will be selected that verifiably represent the participant quotes contained within each theme, and these will be checked by the research team as a form of peer debriefing to identify blind spots and adjusted when found to be dissimilar.

Sequential analysis will be conducted by comparing the themes from interviews to overall changes in participant scores on psychometric measures of anhedonia from preintervention to postintervention assessments to identify any correspondences. Themes will also be compared to any changes in activity across reward processing regions during reward fMRI tasks and targeted music listening evident from preintervention to postintervention assessments that correspond with patterns of congruence in the psychometric data.

Feasibility will be documented based on verbal feedback following fMRI data acquisition and in the final interview. Anything beyond individual preferences will be used to inform necessary changes to the protocol.

## Results

The study received internal funding support from the University of Melbourne in September 2024. MRI paradigm development and testing with 5 healthy volunteers commenced in September 2024 and was finalized in July 2025. Participant recruitment began in July 2025, and data collection commenced in August 2025 and was concluded by mid-2026. As of September 2026, 13 participants have been recruited, and data will be analyzed by the end of 2026, with results expected to be published in 2027.

## Discussion

### Principal Findings

Music therapy has demonstrated feasibility and effectiveness in reducing negative symptoms and/or enhancing quality of life in a range of chronic mental health and physical health conditions. This study outlines a unique protocol for a mixed methods pre-post pilot test that is based on findings from 3 prior case studies using the same intervention, which resulted in improved outcomes among all participants. It aims to investigate whether music therapy reduces anhedonia symptomatology and to explore the interaction between a combination of biophysiological, neuropsychological, and sociobehavioral mechanisms of improvement. Biophysiological mechanisms will be investigated through a comparison of musical pleasure in healthy volunteers and the intervention group. Neuropsychological mechanisms will be investigated using a comparison of preintervention and postintervention test scores on psychometric measures of anhedonia. Sociobehavioral outcomes will be explored through evaluative interviews that probe the importance of the regular therapeutic care and support provided.

No other studies have integrated data to explore the effect of music therapy on anhedonia. Only 1 other study has been identified that reports improvements in anhedonia symptoms, which was based on a similar program that used mindfulness as the key intervention rather than music therapy [8]. We hypothesize that musical pleasure may serve as a gateway to the reactivation of the pleasure system and propose that regular, guided, personally selected pleasure-inducing music activities can promote increased limbic activity, which may be one mechanism through which music therapy reduces anhedonia symptomatology. Currently, this is a 4-month process involving weekly meetings with the guiding therapist and daily engagement with pleasure-inducing music-based activities. This process could be changed and/or made more efficient with different populations once the mechanism is better understood.

### Limitations

The lack of a control group and the small sample size are limitations of the study design. A control group will be included in subsequent studies, once we have determined whether the intervention increases hedonic capacity among individuals with anhedonia and whether any congruences across psychometric results and interview data are identified. This pilot will also enable us to test whether the musical pleasure fMRI paradigm can produce results indicative of reward pathways in those with anhedonia. Nevertheless, it will remain challenging to blind both participants and researchers. The enhanced sensitivity and specificity of fMRI data acquired on the 7T MAGNETOM Plus MRI system may help mitigate the limitations associated with the small number of participants.

There will be some flexibility in the delivery of the intervention since therapeutic integrity is challenged by an overly fixed intervention manual that does not respond to presenting needs. We have published the music therapy intervention [22] that addresses validity and replicability by transparently outlining four categories of principles: (1) unique and essential, (2) essential but not unique, (3) acceptable but not necessary, and (4) not acceptable (proscribed), as recommended in previous music therapy studies [59]. This framework has been used in this study and in additional case studies.

### Conclusions

It is hoped that the results of this pilot study will contribute to an understanding of the benefits of music therapy for people with anhedonia, as well as whether musical pleasure mediates outcomes of music therapy more broadly, particularly in chronic mental and physical health conditions. Greater knowledge of both of these possibilities could result in improved quality of life for people living with chronic ill health, particularly for those with anhedonia, which is associated with poorer treatment adherence and worse long-term outcomes [60] and for which few helpful interventions have been identified to date. This could lead to further exploration and understanding of the role of creative arts therapies and leisure activities in supporting people living with chronic conditions more broadly.

## Supplementary material

10.2196/90667 Multimedia Appendix 1 Exit interview focus areas.

## Abbreviations

BOLD: blood oxygenation level–dependent
DARS: Dimensional Anhedonia Rating Scale
fMRI: functional magnetic resonance imaging
GRAPPA: generalized autocalibrating partially parallel acquisition
MBCIU: Melbourne Brain Centre Imaging Unit
MDD: major depressive disorder
MP2RAGE: magnetization-prepared 2 rapid acquisition gradient echoes
MRI: magnetic resonance imaging
PET: positron emission tomography
RQ: research question
SHAPS: Snaith-Hamilton Pleasure Scale
SPM12: Statistical Parametric Mapping version 12
