# Supplementary material for: Music Therapy for Reduction of Anhedonia: Protocol for a Sequential Mixed Methods Pre-Post Pilot Study
Source: JMIR Res Protoc. 2026 Sep 23;15:e90667. doi: 10.2196/90667 (PMC13600583; doi:10.2196/90667)
Supplement: Supplementary file 1 [file resprot-v15-e90667-s001.docx]

Appendix A

**Exit Interview – Focus Areas**

-Reflecting back on why you joined the study, how are you doing now? What did participation in the study contribute to that?

-How much did your recovery feel achieved by your own intentional efforts and how much did you rely on the music therapist for guidance – did this change over time?

-What aspects of the study did you find most beneficial? What was different to what you expected?

-This study is about pleasure. How would you describe your relationship to pleasure now? Can you compare that to how you related to pleasure when we started this process?

-What conditions did you need for you to be able to access the pleasure?

-Can you describe what pleasure feels like for you at all?

-What was the strength of connection between musical pleasure and reconnecting with other pleasures?
